# Large for Gestational Age and Risk for Academic Delays and Learning Disabilities: Assessing Modification by Maternal Obesity and Diabetes

**DOI:** 10.3390/ijerph17155473

**Published:** 2020-07-29

**Authors:** Kathleen O’Connor Duffany, Katharine H. McVeigh, Heather S. Lipkind, Trace S. Kershaw, Jeannette R. Ickovics

**Affiliations:** 1 Department of Social and Behavioral Sciences, Yale School of Public Health, New Haven, CT 06410, USA; trace.kershaw@yale.edu (T.S.K.); jeannette.ickovics@yale-nus.edu.sg (J.R.I.); 2 Division of Family and Child Health, New York City Department of Health and Mental Hygiene, New York, NY 10013, USA; tmcveigh@health.nyc.gov; 3 Department of Obstetrics, Gynecology, and Reproductive Science, Yale University School of Medicine, New Haven, CT 06510, USA; heather.lipkind@yale.edu; 4 Division of Social Sciences, Yale-NUS College, Singapore 138527, Singapore

**Keywords:** gestational diabetes, large for gestational age (LGA), maternal obesity, academic delays, special education

## Abstract

The objective of this study was to examine academic delays for children born large for gestational age (LGA) and assess effect modification by maternal obesity and diabetes and then to characterize risks for LGA for those with a mediating condition. Cohort data were obtained from the New York City Longitudinal Study of Early Development, linking birth and educational records (*n* = 125,542). Logistic regression was used to compare children born LGA (>90th percentile) to those born appropriate weight (5–89th percentile) for risk of not meeting proficiency on assessments in the third grade and being referred to special education. Among children of women with gestational diabetes, children born LGA had an increased risk of underperforming in mathematics (ARR: 1.18 (95% CI: 1.07–1.31)) and for being referred for special education (ARR: 1.18 (95% CI: 1.02–1.37)). Children born LGA but of women who did not have gestational diabetes had a slightly decreased risk of academic underperformance (mathematics-ARR: 0.94 (95% CI: 0.90–0.97); Language arts-ARR: 0.96 (95% CI: 0.94–0.99)). Children born to women with gestational diabetes with an inadequate number of prenatal care visits were at increased risk of being born LGA, compared to those receiving extensive care (ARR: 1.67 (95% CI: 1.20–2.33)). Children born LGA of women with diabetes were at increased risk of delays; greater utilization of prenatal care among these diabetic women may decrease the incidence of LGA births.

## 1. Introduction

Maternal obesity and diabetes during pregnancy increase risks for maternal and infant morbidity [1,2,3,4,5,6,7]. Among these risks, being born large for gestational age (>90th percentile weight for gestational age) is associated with adverse perinatal outcomes including shoulder dystocia and birth injury [6]. Children born large for gestational age (LGA) of obese or diabetic women also are at increased risk of metabolic syndrome in childhood [8]. The long-term delays associated with being born LGA of women with and without metabolic conditions have not been well-studied.

Paulson, Mehta, Sokol and Chauhan examined the association between LGA long-term cognitive differences and reported no difference when children were compared to those born appropriate weight for gestational age (5–89th percentile) [9]. Khambalia, Algert, Bowen, Collie and Roberts reported a higher percentage of children born LGA at term meeting national reading standards compared to those born appropriate weight at term [10]. However, these studies did not differentiate and report by maternal risk factors such as maternal obesity and diabetes during pregnancy. This may be important as these maternal conditions may differentially affect fetal cognitive development and later academic achievement.

As mechanisms for large fetal growth are diverse [11], we hypothesized that risks for delays associated with being born LGA may be modified by maternal conditions in which aberrant fetal growth may be accelerated by a disturbed metabolic milieu, specifically in pregnancies complicated by obesity and diabetes and this disturbed milieu may alter cognitive development. While early childhood environment, nutrition and socioeconomic and cultural factors play a role in shaping neurodevelopment and educational attainment, further exploration of specific mechanisms in the fetal environment impacting these outcomes is critical. Using a large, US-based cohort, the aim of this study was to assess risks for long-term delays for children born LGA as compared to children born appropriate weight for gestational age and to assess effect modification by maternal obesity and diabetes. When an association was identified, we sought to characterize the maternal and perinatal differences between the children born large versus appropriate weight for gestational age. We hypothesize that children born LGA of women with a metabolic condition would be at increased risk of adverse perinatal outcomes. We also hypothesize that poor utilization of prenatal care may be associated with children being born LGA and therefore a potential area for intervention.

## 2. Materials and Methods

### 2.1. Population

Cohort data were obtained from the NYC Longitudinal Study of Early Development data warehouse that contains records from the NYC Department of Health and Mental Hygiene of all births in NYC from 1994–2004 linked with the NYC Department of Education’s records of children that attended NYC public schools. Data were obtained from the Longitudinal Study of Early Development data warehouse, a compilation of linked deidentified data from the NYC Department of Health and Mental Hygiene and the NYC Department of Education. This database comprises linked data from birth records and school reports, including achievement scores on the standards-based mathematics and English language arts assessments administered in the third grade and information on whether the child was referred for special education at any time. For our study, the inclusion criteria required the availability of birth record data and third-grade test score data. Children born of a singleton pregnancy to a mother 18–45 years of age were included in this study. To eliminate other causes of neurodevelopmental delays, children born with a genetic or congenital anomaly, to a mother with rubella or a sexually transmitted disease, or born less than 32 weeks gestational age were excluded.

The study was limited to children born large or appropriate weight for gestational age; children born small for gestational age (<10th percentile weight for gestational age) were removed from the analytical cohort.

This study was approved by the Department of Health and Mental Hygiene Institutional Review Board. All data were deidentified.

### 2.2. Measures

Gender-specific US-standard birth weights for gestational age reference values derived by Oken et al. were used to create the weight for gestational age variables using birth weight and gestational age, as used in other studies [9,12]. Children born at the 10–90th percentile weight for gestational age were coded as an appropriate weight for gestational age (AGA) and greater than the 90th percentile were coded as LGA.

Maternal obesity was reported as ≥200 lb prepregnancy as done in other studies when height data were not available to compute BMI [13,14]. As a reference, at ≥200 lb, women shorter than 5’9”, which represents 95% of the population, would have a BMI of ≥30 kg/m^2^ which is obese [15,16]. Maternal diabetes was assessed as two separate conditions available in this dataset: pregestational and gestational diabetes. When both conditions were indicated, the condition was recoded as pregestational diabetes, only.

Outcomes assessed included children meeting proficiency on the third grade standards-based mathematics and English language arts assessments and being referred for special education at any time. Nationally in the US, statewide educational assessments are required as part of the No Child Left Behind Act of 2001, Public Law 107–110, with the goal for all students to reach proficiency on each subject-based assessment [17]. The development process for these subject-based standards assessments includes a rigorous process at the state-level including assessment of content and construct validity [18]. Student performance scores on these assessments are reported on a four-point scale. A score of 1 or 2 indicates the student did not meet proficiency; conversely, a score of 3 or 4 indicates the student met or exceeded proficiency. We assessed the bivariate outcome: met/did not meet proficiency on the mathematics assessment and met/did not meet proficiency on the English Language arts assessment.

Covariates included maternal race/ethnicity, age, education, country of origin, marital status, insurance payer, parity, tobacco, alcohol or drug use during pregnancy, excessive gestational weight gain, infant sex and year of birth. Covariates were chosen based on the theoretical relevance of each variable. Variable coding was identical to our previous study and noted in Table 1 (in Results section).

If the effect of being born LGA in relation to delays differed depending on whether the children’s mothers had a particular metabolic condition during pregnancy, perinatal outcomes for the group were characterized and the association between maternal prenatal care utilization and being born LGA within the group was assessed. Perinatal outcomes included dichotomous variables (yes/no) for maternal chronic hypertension, pre-eclampsia, eclampsia, preterm birth, need for neonatal intensive care unit, abnormal metabolic condition of newborn, anemia, seizure, intubation of newborn and birth injury.

The Kotelchuck Adequacy of Prenatal Care Utilization (APNCU) Index was used to assess prenatal care utilization. The APNCU Index, detailed elsewhere [19], is a summary index consisting of two components: Adequacy of Initiation of Prenatal Care and Adequacy of Received Visits. Each component is reported on a four-point scale. The Adequacy of Initiation of Prenatal Care scale relates to the month prenatal care was initiated (Adequate-Plus = months 1–2, Adequate = months 3–4, Intermediate = months 5–6 and Inadequate = months 7–9 or no care at all); the Adequacy of Received Visits scale is based on the percent of visits received of those expected in an uncomplicated pregnancy adjusted for gestational age of birth (Adequate-Plus = 110% or more; Adequate = 80–109%, Intermediate = 50–79%; Inadequate = 0–49%). Relative risks were assessed for all four categories for each scale and for a three-category model, collapsing Inadequate and Intermediate levels into one category.

### 2.3. Statistical Analysis

Chi-square analyses were used to report bivariate associations for maternal and infant characteristics. Relative risk analyses were conducted to compare risks for children born large and appropriate weight for gestational age for three outcomes: not meeting proficiency on the third grade standards-based mathematics assessment, not meeting proficiency on the third grade standards-based English language arts assessment and referred for special education at any time. Then, effect modification by maternal obesity, pregestational diabetes and gestational diabetes was assessed using an interaction term approach [20], analyzing interaction as well as relative risks in the presence and absence of these potential modifiers. Analyses included modeling unadjusted, partially adjusted and fully adjusted relative risk models using the GENMOD procedure with a binomial distribution. For all risk analyses, a modified Poisson was employed when binomial models did not converge [21,22]. Sensitivity analyses assessed fully adjusted risk models controlling for preterm birth.

When obesity, pregestational diabetes or gestational diabetes modified the effect of LGA, chi-square analyses were used to characterize maternal, neonatal and prenatal care differences between children born large and appropriate weight for gestational age, among children of women with the effect modifying condition. For these analyses, children with missing or incomplete data for the prenatal care variables were removed. Relative risks were calculated for maternal prenatal care utilization for children born LGA within the effect modifying group. SAS 9.3 (SAS Institute Inc., Cary, NC, USA) was used for all analyses.

### 2.4. Ethical Statement

The Longitudinal Study of Early Development (IRB No. 08-046) was approved by the NYC Department of Health and Mental Hygiene in June 2008 (approval letter dated 11 July 2008 and initial approval period expiring 22 June 2009). The Longitudinal Study of Early Development (LSED) dataset was created by linking administrative records, compiling analytic files and then removing and permanently destroying all personally identifying information. Because the dataset was deidentified, no consent was required.

## 3. Results

Table 1 reports characteristics of the study cohort. Being born LGA evidenced a small but significant protective effect on not meeting proficiency on the standards-based assessments (unadjusted LGA vs. AGA: mathematics—23.4% vs. 24.6%, *p* = 0.02; English language arts—39.9% vs. 41.3%, *p* = 0.01). In fully adjusted relative risk models, compared to children born appropriate weight for gestational age, children born LGA had a lower risk of not meeting proficiency on the mathematics (ARR: 0.96 (95% CI: 0.92–0.99)) and English language arts (ARR: 0.97 (95% CI: 0.95–0.99)) assessments but this risk approached nonsignificance (Table 2).

**Table 1:** Characteristics of cohort by LGA and AGA (*n* = 108,348) ^a^.

Characteristic	Total 108,348		LGA *n* = 8634 (8.0%)		AGA *n* = 99,714 (92.0%)		*p*-Value
	*n*	%	*n*	%	*n*	%	
Maternal race/ethnicity							<0.001
Non-Hispanic black	33,931	31.3	2474	28.7	31,457	31.6	
Non-Hispanic white	20,189	18.6	1959	22.7	18,230	18.3	
Hispanic	41,344	38.2	3469	40.2	37,875	38.0	
Asian	12,422	11.5	704	8.2	11,718	11.8	
Other	462	0.4	28	0.3	434	0.4	
Maternal age							<0.001
18 < 20	8461	7.8	406	4.7	8055	8.1	
20 < 35	82,230	75.9	6435	74.5	75,795	76.0	
35+	17,657	16.3	1793	20.8	15,864	15.9	
Maternal education							0.01
<high school graduate	30,627	28.3	2339	27.1	28,288	28.4	
Maternal nativity							0.03
Foreign born	55,403	51.1	4513	52.3	50,890	51.0	
Marital status							<0.001
Not married	58,462	54.0	4320	50.0	54,142	54.3	
Insurance payer: Medicaid							<0.001
Yes	64,691	59.7	4836	56.0	59,855	60.0	
Parity							<0.001
Nulliparous	42,538	39.3	2601	30.1	39,937	40.1	
Tobacco use ^b^							<0.001
Yes	5433	5.0	276	3.2	5157	5.2	
Alcohol use ^b^							0.12
Yes	407	0.4	24	0.3	383	0.4	
Drug use ^b^							<0.001
Yes	1043	1.0	37	0.4	1006	1.0	
Infant Sex							0.22
Male	53,668	49.5	4222	48.9	49,446	49.6	
Gestational weight gain							<0.001
Excessive	22,509	20.8	3101	35.9	19,408	19.5	
Obesity (≥200 lb)							<0.001
Yes	8214	7.6	1315	15.2	6899	6.9	
Pregestational diabetes							<0.001
Yes	358	0.3	71	0.8	287	0.3	
Gestational diabetes							<0.001
Yes	4598	4.2	754	8.7	3844	3.9	

Note: AGA, appropriate weight for gestational age (10–90th percentile); LGA, large for gestational age (>90th percentile). ^a^ Based on Oken curve (BMC Pediatr. 2003). ^b^ Maternal behaviors during pregnancy. ^c^ Gestational weight gain was considered excessive if women weighing less than 200 lbs gained more than 40 lb or if women weighing 200 lb or more gained more than 25 lb. These weight gain cut-points represent the most weight a woman falling within each dichotomous weight category (underweight/normal/overweight and overweight/obese) are recommended to gain. [23].

### 3.1. Assessing Effect Modification by Maternal Obesity or Diabetes

In this cohort (*n* = 108,348), 8% of children were born LGA. Being born LGA was more prevalent among children of women with obesity and diabetes: 16% of children born to women who were obese, 19.8% of children born to women with pregestational diabetes and 16.4% of children born to women with gestational diabetes were born LGA.

#### 3.1.1. Obesity

There was no significant effect modification by maternal obesity.

#### 3.1.2. Pregestational Diabetes

Among children born to women with pregestational diabetes, comparing children born LGA or appropriate weight for gestational age, there was no significant difference in percentage meeting proficiency on the mathematics or English language arts assessments (Mathematics LGA vs. AGA: 35.2% vs. 32.4%; *p* = 0.65; English language arts-LGA vs. AGA: 54.9% vs. 45.6%; *p* = 0.16). Likewise, no significant increased risk was found for children born LGA to women with pregestational diabetes (Mathematics ARR: 1.08 (0.78–1.50); English language arts ARR: 1.14 (0.92–1.41); special education ARR: 0.94 (0.63–1.38)).

#### 3.1.3. Gestational Diabetes

Comparing risks for children born large versus appropriate weight for gestational age, gestational diabetes significantly modified the effect on each outcome. Among children born to women with gestational diabetes, children born LGA were at increased risk of not meeting proficiency on the mathematics assessment (ARR: 1.18 (95% CI: 1.07–1.31)). Conversely, among children of women who did not have gestational diabetes, being born LGA had a protective effect on not meeting proficiency on assessments (mathematics ARR: 0.94 (95% CI: 0.90–0.97); English language arts ARR: 0.96 (95% CI: 0.94–0.99)) compared to children born appropriate weight for gestational age. Additionally, children born LGA evidenced an increased risk of being referred for special education if the mother had gestational diabetes (ARR: 1.18 (95% CI: 1.02–1.37)) but no increased risk if the mother did not have gestational diabetes (ARR: 0.96 (95% CI: 0.92–1.01)). The results are detailed in Table 3.

### 3.2. Children of Women with Gestational Diabetes: Characterizing Children Born LGA

Associations between maternal characteristics as well as adverse maternal and neonatal conditions related to gestational diabetes and being born LGA are detailed in Table 4. Among children of women with gestational diabetes, being born LGA was associated with the mother not being married, multiparity, maternal obesity, excessive gestational weight gain and a prior sibling born 4000 g or more. No associations were found between maternal chronic hypertension, preeclampsia, eclampsia and being born LGA. Being born LGA was not associated with being admitted to the neonatal intensive care unit. Among women with gestational diabetes, no infant born LGA had a metabolic disorder at birth, anemia, a seizure or birth injury and prevalence of intubation was similar for infants born large or appropriate weight for gestational age.

Among children born to women with gestational diabetes, children of women receiving Inadequate levels of expected prenatal care visits were 67% more likely than children of women receiving Adequate-Plus levels of visits to be born LGA (ARR: 1.67 (95% CI: 1.20–2.33)). Furthermore, the summary APNCU Index indicated an increased risk of being born LGA for children of women in the Adequate and Inadequate/Intermediate categories compared to children of women who were in the Adequate-Plus category (Adequate ARR: 1.25 (95% CI: 1.05–1.48); Inadequate/Intermediate ARR: 1.21 (95% CI: 1.02–1.44)).

## 4. Discussion

This study explored the effect of being born LGA on academic delays and potential moderation by maternal diabetes and obesity and characterized the risks for LGA for children of mothers with the mediating condition. We found that children born LGA evidenced similar or significantly better assessment outcomes than children born appropriate weight for gestational age, unless the mother had gestational diabetes. For children of women without gestational diabetes being born LGA was protective for not meeting proficiency on the standards-based assessments, while for children of women with gestational diabetes being born LGA was a risk factor for not meeting proficiency on the mathematics assessment and for being referred for special education.

While LGA is a notable adverse outcome for mother and infant in the perinatal period, our study suggests that being born LGA may be associated with positive long-term outcomes in some pregnancies but may be an indicator for poor long-term learning and neurodevelopmental outcomes in others. The mechanisms driving the differences seen for children born LGA is an important consideration. LGA may be a result of genetic factors or an adverse fetal environment. Explaining the association between gestational diabetes and LGA, the Pederson Hypothesis promotes that hyperglycemia in the mother leads to hyperinsulin in the fetus and accelerated fetal growth [24]. In addition to fetal growth, the fluctuations of insulin and glucose lead to hypoxia-ischemia, hypoglycemia, and iron deficiency, which may adversely affect the developing brain, specifically the hippocampus [25].

In addition to suggesting that delays for children of diabetic women may be associated with alteration in brain development due to the milieu in utero, it has been suggested that long-term delays may also be a function of adverse perinatal events associated with diabetic pregnancies [25,26]. In our study, among women with gestational diabetes, neonatal anemia, metabolic condition of the newborn, infant need for intubation, seizure, birth injury, being born preterm or need for neonatal intensive care unit were not more common among LGA infants as compared to infants born appropriate weight for gestational age. Therefore, in our study, we cannot attribute the increased risk of delays seen for LGA children of women with gestational diabetes to an increased rate of these events.

Children of women with an Inadequate/Intermediate or Adequate score on the summary APNCU Index evidenced an increased risk of being born LGA. In this cohort, scoring Adequate-Plus on the APNCU Index and the Adequacy of Received Visits Index may be indicative of better diabetic care and tighter glycemic control, leading to a more appropriate fetal growth rate. This is supported by studies reporting that an intensification of diabetic care during pregnancy reduces rates of LGA and fetal macrosomia to levels seen in nondiabetic populations [27,28,29].

### 4.1. Strengths and Limitations

Our study assessed a large US-based cohort, assessed children through the third grade on multiple measures and considered effect modification by maternal obesity and diabetes, contributing to the recent literature on long-term risks for children born LGA. As far as we know, our study is the first US-based cohort study to assess long-term academic outcomes and the need for special education for children born LGA with effect modification by maternal obesity and diabetes, maternal conditions at high-risk of an LGA birth.

There is a need for additional studies on long-term neurodevelopmental, learning and academic outcomes of children born LGA. While Paulson et al. reported no significant difference between infants born large and appropriate weight for gestational age at nine months, two years, prekindergarten and kindergarten [9], three other studies, two which focused on results for children born small for gestational age, support our findings that LGA may be protective in some general populations. A study in the Republic of Belarus assessing the association of weight for gestational age and IQ in 6.5-year-old children found a higher mean full-scale and verbal IQ score for children born LGA compared to children born small for gestational age (<10th percentile) and to children at the 10–50th percentile appropriate weight [30]. A cohort study in the United Kingdom assessing risks for specific causes for special education need by gestational age and birth weight percentile found being born at the 91–97th percentile significantly reduced risk of intellectual impairment when compared to children born at the 21–80th percentile reference range (ARR: 0.88 (CI: 0.79–0.98)) [31]. Additionally, a study in Australia found children born LGA at term were more likely to meet reading standards compared to an appropriate weight counterpart [10]. Of note, these prior studies did not assess results stratified by maternal obesity and diabetes. Our study also assessed these effects controlling for preterm birth, yet no differences were found. Our study highlights that, compared to an appropriate weight for gestational age, being born LGA may decrease the risk of academic and learning delays within some populations and increase risk within others, a discrepancy that may be missed when assessing LGA risks without stratification by maternal conditions. With high rates of obesity and diabetes in young women and the association between obesity and diabetes in pregnancy and higher rates of LGA births, this is of particular public health interest as the percent of LGA infants is likely to increase.

We were limited by the data available from birth certificates. As noted, using BMI as the measure to identify maternal obesity would be preferable, but height data were not available to compute BMI. Additionally, although our study controlled for many maternal and birth characteristics, our study was unable to control for socioeconomic status. Although we controlled for maternal Medicaid use and maternal education, which may serve as proxies for this unavailable measure, residual confounding may remain. We were unable to control for other potential confounders such as nutrition status of child or parental status. Further, other factors may influence or mediate risk such as maternal intelligence [32], metabolic syndrome [8] or obesity in childhood [33], physical activity during pregnancy [34,35] and breastfeeding [36]. Again, these data were not available in birth or educational records but would be of interest. Additionally, misclassification or under-reporting of some conditions or complications in birth records may have occurred. While patients with rubella and other sexually transmitted infections were excluded, other infections that are associated with poor neurodevelopment (such as CMV) were not evaluated for in this dataset, and, therefore, were not excluded.

In assessing prenatal care utilization, we found Adequate-Plus levels to be associated with better outcomes on two of the indices, yet some studies using the APNCU Index report a U-shaped association with poor outcomes for women in the Adequate-Plus category [37,38]. As the APNCU Index and the Adequacy of Received Visits Index were created based on the number of visits expected in uncomplicated pregnancies, it may underestimate the adequate number of prenatal care visits for women with medical risks such as diabetes and the Adequate-Plus category may better represent standard or adequate care for women with diabetes [19] and as such, in our cohort, found to be associated with better outcomes within this group.

### 4.2. Future Directions

Future studies could seek to address additional factors including birth order, maternal weight loss between pregnancies and different levels of BMI which was unavailable for this study [39]. Studies may also explore effects to differential exposures to medications on LGA and neurodevelopmental and general neonatal outcomes.

Follow-up studies may be able to assess the risks for children of women with pregestational diabetes; our study was not powered to conclusively compute these risks. As the prevalence of pregestational diabetes in women of childbearing age will likely increase in future birth cohorts [40,41], assessing the risks for children born LGA of women with pregestational diabetes may be possible and of interest.

Additionally, studies assessing prenatal care strategies for women with gestational diabetes, including nutritional therapy, may look to assess the impact on LGA rates as well as delays in children [42]. Understanding the potential for mitigation of delays is an important next step. Large-scale public health interventions taking a preventative approach, such as lifestyle interventions and other programs addressing overweight and obesity before pregnancy may reduce the rate of gestational diabetes and the resulting delays seen in children born LGA of gestational diabetes [43]. Studies should also explore other discrepant characteristics between children born LGA and AGA (e.g., head-size, microcephaly, hearing or vision loss, history of oxygen use) noting potential mechanisms and areas for intervention.

Lastly, future replication studies should be conducted in other populations beyond public school students in large US cities, as the generalizability of this study is limited by those parameters. Studies based in diverse low- and middle-income countries, as well as replication studies assessing outcomes by country of birth, race/ethnicity and by population density (rural/urban), should be considered [44].

## 5. Conclusions

This study suggests that being born LGA may have a protective effect on some children and a detrimental effect on children born LGA to women with gestational diabetes. Among children of women with gestational diabetes, being born LGA was associated with poorer academic outcomes than being born appropriate weight for gestational age; however, achieving an appropriate number of prenatal care visits and increasing utilization of prenatal care among women with gestational diabetes may reduce the number of children born LGA. While LGA is considered to be a risk of adverse perinatal events, long-term cognitive and neurodevelopmental effects associated with being born LGA to women with various conditions have not been well-studied. The long-term risks reported in this study may support interventions to reduce rates of LGA in pregnancies complicated by gestational diabetes. Future studies are needed in other cohorts to confirm these findings.

## Figures and Tables

**Table 2:** Relative risks for long-term academic outcomes and delays for children born LGA (*n* = 108,348).

Outcome	Category	Unadjusted RR	Fully Adjusted RR ^a^
Did not meet proficiency on mathematics ^b^	AGA LGA	Reference 0.95 (0.92–0.99)	Reference 0.96 (0.92–0.99)
Did not meet proficiency on English language arts ^b^	AGA LGA	Reference 0.97 (0.94–0.99)	Reference 0.97 (0.95–0.99)
Referred for special education	AGA LGA	Reference 1.01 (0.97–1.06)	Reference 0.98 (0.94–1.03)

Note: AGA, appropriate weight for gestational age (10–90th percentile); LGA, large for gestational age (>90th percentile). ^a^ The fully adjusted models controlled for the following covariates: maternal race/ethnicity, age, education, nativity, marital status, Medicaid status, parity, maternal obesity, pregestational and gestational diabetes, tobacco, alcohol and drug use during pregnancy, excessive gestational weight gain, infant sex and year of birth. Similar results were found in partially adjusted models that controlled for all covariates with the exception of maternal obesity and diabetes (with a slightly less protective effect). Models also controlling for preterm birth (32–36 weeks versus 37 weeks or more) provided similar results. Maternal chronic hypertension was not controlled for as it was not significant in models and its inclusion did not significantly change results. ^b^ The bivariate outcome used for not meeting proficiency on standards-based assessments is based on a common educational indicator which denotes failing based on proficiency scores; scoring a 1 or 2 indicates the child did not meet proficiency levels expected at that grade, while scoring a 3 or 4 indicates meeting expected proficiency and above.

**Table 3:** Relative Risks and Effect Modification for those born LGA compared to those born AGA by Maternal Gestational Diabetes Status (*n* = 108,348).

Category	Unadjusted			Fully Adjusted		
	Infants of Mothers without Gestational Diabetes	Infants of Mothers with Gestational Diabetes		Infants of Mothers without Gestational Diabetes	Infants of Mothers with Gestational Diabetes ^1^	
Did not meet proficiency on Mathematics						
AGA	Reference	Reference		Reference	Reference	
LGA	0.92 (0.88–0.96)	1.28 (1.14–1.45)	ⱡ *	0.94 (0.90–0.97)	1.18 (1.07–1.31)	ⱡ *
Did not meet proficiency on English Language Arts						
AGA	Reference	Reference		Reference	Reference	
LGA	0.95 (0.92–0.98)	1.13 (1.03–1.23)	ⱡ *	0.96 (0.94–0.99)	1.05 (0.97–1.13)	ⱡ
Recommended for Special Education						
AGA	Reference	Reference		Reference	Reference	
LGA	0.99 (0.94–1.04)	1.26 (1.08–1.47)	ⱡ	0.96 (0.92–1.01)	1.18 (1.02–1.37)	ⱡ

ⱡ Effect Modification. * Qualitative interaction. 1: The fully adjusted models controlled for the following covariates: maternal race, nativity, education, marital status, Medicaid status, parity, maternal obesity and diabetes, alcohol, drug and tobacco use during pregnancy, excessive weight gain during pregnancy, and infant gender. Similar results were found in partially adjusted models that controlled for all covariates with the exception of maternal obesity and diabetes (with a slightly less protective effect). Models also controlling for preterm birth (32–36 weeks vs. 37 weeks or more) provided similar results.

**Table 4:** Characteristics of children of women with gestational diabetes by LGA and AGA (*n* = 4113) ^a^.

Characteristic	Total		LGA *n* = 667 (16.2%)		AGA *n* = 3446 (83.8%)		*p*-Value
	*n*	%	*n*	%	*n*	%	
Maternal race/ethnicity							<0.001
Non-Hispanic black	1239	30.1	213	31.9	1026	29.8	
Non-Hispanic white	601	14.6	106	15.9	495	14.4	
Hispanic	1553	37.8	278	41.7	1275	37.0	
Asian	682	16.6	67	10.0	615	17.9	
Other	38	0.9	3	0.5	35	1.0	
Maternal age							0.84
18 < 20	72	1.8	10	1.5	62	1.8	
20 < 35	2794	67.9	457	68.5	2337	67.8	
35+	1247	30.3	200	30.0	1047	30.4	
Maternal education							0.10
< HS graduate	1156	28.1	205	30.7	951	27.6	
Maternal nativity							0.28
Foreign born	2458	59.8	386	57.9	2072	60.1	
Marital status							0.04
Not married	1994	48.5	348	52.2	1646	47.8	
Insurance payer: Medicaid							0.99
Yes	2503	60.9	406	60.9	2097	60.9	
Parity							<0.001
Nulliparous	1285	31.2	148	22.2	1137	33.0	
Tobacco use during pregnancy							0.38
Yes	174	4.2	24	3.6	150	4.4	
Alcohol use during pregnancy							-
Yes	8	0.2	2	0.3	6	0.17	
Drug use during pregnancy							-
Yes	13	0.3	1	0.4	12	0.4	
Infant sex							0.13
Male	2065	50.2	317	47.5	1748	50.7	
Gestational weight gain							<0.001
Excessive	846	20.6	221	33.1	625	18.1	
Obesity (≥200 lb)							<0.001
Yes	667	16.2	161	24.1	506	14.7	
Prior sibling born 4000+ g							<0.001
Yes	49	1.2	21	3.2	28	0.8	
Maternal and neonatal conditions/events							
Maternal chronic hypertension							0.84
Yes	122	3.0	19	2.9	103	3.0	
Pre-eclampsia							0.76
Yes	150	3.7	23	3.5	127	3.7	
Eclampsia							
Yes	5	0.12	0	-	5	0.15	
Preterm (32–36 weeks)							0.001 ^b^
Yes	362	8.8	37	5.6	325	9.4	
Needed NICU							0.44
Yes	556	15.9	97	17.0	459	15.7	
Missing (*n* = 617 (15.0%))			96	14.4	521	15.1	
Abnormal metabolic condition of newborn							
Yes	0	0					
Missing (*n* = 675 (16.4%))			106	15.9	569	16.5	
Infant anemic at birth							
Yes	3	0.1	0	0	3	0.1	
Seizure of newborn							
Yes	3	0.1	0	0	3	0.1	
Intubation of newborn							
Yes	19	0.5	3	0.5	16	0.5	
Birth injury							
Yes	1		0		1		
Prenatal care							
Trimester of first prenatal care visit							0.90
≤91 days	2801	68.1	459	68.8	2342	68.0	
92–189 days	1137	27.6	183	27.4	954	27.7	
190+ days	159	3.9	23	3.5	136	3.9	
No prenatal care	16	0.4	2	0.3	14	0.4	

AGA, appropriate weight for gestational age (10–90th percentile); LGA, large for gestational age (>90th percentile). ^a^ 485 subjects had missing or incomplete data for prenatal care variables. Significance did not change when assessing all 4598 children of women with gestational diabetes prior to removing subjects missing prenatal care data. ^b^ Infants born LGA were significantly less likely to be born preterm.

## References

[1] Athukorala C., Rumbold A.R., Willson K.J., Crowther C.A. (2010). The risk of adverse pregnancy outcomes in women who are overweight or obese. BMC Pregnancy Childbirth.

[2] Meur S., Mann N.P. (2007). Infant outcomes following diabetic pregnancies. Paediatr. Child Health.

[3] Schwartz R., Teramo K.A. (2000). Effects of diabetic pregnancy on the fetus and newborn. Semin. Perinatol..

[4] Scott-Pillai R., Spence D., Cardwell C., Hunter A., Holmes V.A., Cardwell C.R. (2013). The impact of body mass index on maternal and neonatal outcomes: A retrospective study in a UK obstetric population, 20042–011. BJOG Int. J. Obstet. Gynaecol..

[5] Weindling A.M. (2009). Offspring of diabetic pregnancy: Short-term outcomes. Semin. Fetal Neonatal Med..

[6] Weintrob N., Karp M., Hod M. (1996). Short- and long-range complications in offspring of diabetic mothers. J. Diabetes Its Complicat..

[7] Liu L., Ma Y.-N., Wang N., Lin W., Liu Y., Wen D. (2019). Maternal body mass index and risk of neonatal adverse outcomes in China: A systematic review and meta-analysis. BMC Pregnancy Childbirth.

[8] Boney C.M. (2005). Metabolic Syndrome in Childhood: Association With Birth Weight, Maternal Obesity, and Gestational Diabetes Mellitus. Pediatrics.

[9] Paulson J.F., Mehta S.H., Sokol R.J., Chauhan S.P. (2014). Large for gestational age and long-term cognitive function. Am. J. Obstet. Gynecol..

[10] Khambalia A.Z., Algert C., Bowen J.R., Collie R.J., Roberts C. (2017). Long-term outcomes for large for gestational age infants born at term. J. Paediatr. Child Health.

[11] Henriksen T. (2008). The macrosomic fetus: A challenge in current obstetrics. Acta Obstet. Gynecol. Scand..

[12] Oken E., Kleinman K., Rich-Edwards J., Gillman M.W. (2003). A nearly continuous measure of birth weight for gestational age using a United States national reference. BMC Pediatr..

[13] Biggio J.R., Chapman V., Neely C., Cliver S.P., Rouse D.J. (2010). Fetal anomalies in obese women: The contribution of diabetes. Obstet. Gynecol..

[14] Dodds L., Fell D.B., Shea S., Armson B.A., Allen A.C., Bryson S. (2010). The Role of Prenatal, Obstetric and Neonatal Factors in the Development of Autism. J. Autism Dev. Disord..

[15] Defining Overweight and Obesity: Adult Body Mass Index. https://www.cdc.gov/obesity/adult/defining.html..

[16] Fryar C.D., Gu Q., Ogden C.L. Anthropometric Reference Data for Children and Adults. USA, 2007–2010. https://www.cdc.gov/nchs/products/series/series11.htm.

[17] Mayers C.M. (2006). Public Law 107–110: No Child Left Behind Act of 2001: Support or Threat to Education as a Fundamental Right?. Education.

[18] New York State Education Department (2017). New York State Testing Program 2017: Technical Report.

[19] Kotelchuck M. (1994). An evaluation of the Kessner Adequacy of Prenatal Care Index and a proposed Adequacy of Prenatal Care Utilization Index. Am. J. Public Health.

[20] Van Ness P.H., Allore H.G. Paper 1953–1: Using the SAS system to investigate effect modification. Presented at the Thirty-First Annual SAS Users Group International Conference.

[21] Spiegelman D. (2005). Easy SAS Calculations for Risk or Prevalence Ratios and Differences. Am. J. Epidemiol..

[22] Zou G. (2004). A modified poisson regression approach to prospective studies with binary data. Am. J. Epidemiol..

[23] National Research Council (2009). Weight Gain during Pregnancy: Re-Examining the Guidelines.

[24] Pedersen J. (1955). Weight and length at birth of infants of diabetic mothers. Eur. J. Endocrinol..

[25] Nold J.L., Georgieff M.K. (2004). Infants of diabetic mothers. Pediatr. Clin. N. Am..

[26] Fraser A., Lawlor D. (2014). Long-term health outcomes in offspring born to women with diabetes in pregnancy. Curr. Diabetes Rep..

[27] Crowther C.A., Hiller J., Moss J.R., McPhee A.J., Jeffries W.S., Robinson J.S. (2005). Effect of Treatment of Gestational Diabetes Mellitus on Pregnancy Outcomes. N. Engl. J. Med..

[28] Landon M.B., Spong C.Y., Thom E., Carpenter M.W., Ramin S.M., Casey B., Wapner R.J., Varner M.W., Rouse D.J., Thorp J.M. (2009). A Multicenter, Randomized Trial of Treatment for Mild Gestational Diabetes. N. Engl. J. Med..

[29] Ogonowski J., Miazgowski T., Czeszyńska M.B., Jaskot B., Kuczyńska M., Celewicz Z. (2008). Factors influencing risk of macrosomia in women with gestational diabetes mellitus undergoing intensive diabetic care. Diabetes Res. Clin. Pract..

[30] Yang S., Platt R.W., Kramer M.S. (2010). Variation in Child Cognitive Ability by Week of Gestation Among Healthy Term Births. Am. J. Epidemiol..

[31] Mackay D., Smith G., Dobbie R., Pell J., Cooper S.-A., Smith G. (2012). Obstetric factors and different causes of special educational need: Retrospective cohort study of 407 503 schoolchildren. BJOG Int. J. Obstet. Gynaecol..

[32] Der G., Batty G.D., Deary I.J. (2006). Effect of breast feeding on intelligence in children: Prospective study, sibling pairs analysis, and meta-analysis. BMJ.

[33] Kantomaa M.T., Stamatakis E., Kankaanpää A., Kaakinen M., Rodriguez A., Taanila A., Ahonen T., Jarvelin M.-R., Tammelin T. (2012). Physical activity and obesity mediate the association between childhood motor function and adolescents’ academic achievement. Proc. Natl. Acad. Sci. USA.

[34] Kim H., Lee S.-H., Kim S.-S., Yoo J.-H., Kim C.-J. (2007). The influence of maternal treadmill running during pregnancy on short-term memory and hippocampal cell survival in rat pups. Int. J. Dev. Neurosci..

[35] Robinson A.M., Bucci D.J. (2013). Physical exercise during pregnancy improves object recognition memory in adult offspring. Neuroscience.

[36] Lundgren M., Tuvemo T. (2008). Effects of being born small for gestational age on long-term intellectual performance. Best Pract. Res. Clin. Endocrinol. Metab..

[37] Debiec K.E., Paul K.J., Mitchell C., Hitti J.E. (2010). Inadequate prenatal care and risk of preterm delivery among adolescents: A retrospective study over 10 years. Am. J. Obstet. Gynecol..

[38] Hale N.L., Glover S., Probst J.C., Liu J., Bennett K.J., Martin A. (2011). Variation in Excessive Fetal Growth across Levels of Prenatal Care among Women with Gestational Diabetes. J. Prim. Care Community Health.

[39] Ziauddeen N., Wilding S., Roderick P.J., Macklon N.S., Alwan N.A. (2019). Is maternal weight gain between pregnancies associated with risk of large-for-gestational age birth? Analysis of a UK population-based cohort. BMJ Open.

[40] Feig D., Hwee J., Shah B.R., Booth G.L., Bierman A.S., Lipscombe L.L. (2014). Trends in Incidence of Diabetes in Pregnancy and Serious Perinatal Outcomes: A Large, Population-Based Study in Ontario, Canada, 1996–2010. Diabetes Care.

[41] Fong A., Serra A., Herrero T., Pan D., Ogunyemi D. (2014). Pre-gestational versus gestational diabetes: A population based study on clinical and demographic differences. J. Diabetes Its Complicat..

[42] Farabi S.S., Rn T.L.H. (2019). Low-Carbohydrate Diets for Gestational Diabetes. Nutrients.

[43] Hanson M., Barker M., Dodd J.M., Kumanyika S., Norris S.A., Steegers E., Stephenson J., Thangaratinam S., Yang H. (2017). Interventions to prevent maternal obesity before conception, during pregnancy, and post partum. Lancet Diabetes Endocrinol..

[44] Ferrara A. (2007). Increasing Prevalence of Gestational Diabetes Mellitus: A public health perspective. Diabetes Care.

